# Safety and preliminary efficacy of the Gam-COVID-Vac vaccine and outcomes of SARS-CoV-2 infection in Russian patients with genitourinary malignancies

**DOI:** 10.1186/s13045-021-01205-z

**Published:** 2021-11-13

**Authors:** Ilya Tsimafeyeu, Maria Volkova, Galina Alekseeva, Maria Berkut, Alexander Nosov, Igor Myslevtsev, Andrey Andrianov, Andrey Semenov, Pavel Borisov, Ruslan Zukov, Vadim Goutnik, Sergey Savchuk, Natalia Dengina, Timur Mitin

**Affiliations:** 1 Kidney Cancer Research Bureau, Mayakovskogo pereulok 2, Moscow, 109147 Russia; 2 N.N. Blokhin National Medical Research Center of Oncology, Moscow, Russia; 3 Pacific State Medical University, Vladivostok, Russia; 4 N.N. Petrov National Medical Research Center of Oncology, St. Petersburg, Russia; 5 St. Alexei Central Clinical Hospital, Moscow, Russia; 6 Ivanovo Regional Oncology Dispensary, Ivanovo, Russia; 7 V.F. Voyno-Yasenetsky Krasnoyarsk State Medical University, Krasnoyarsk, Russia; 8 Medicine 24/7 Clinic, Moscow, Russia; 9 Saint-Petersburg Clinical Scientific and Practical Center for Specialized Types of Medical Care (Oncological), St. Petersburg, Russia; 10 Ulyanovsk Regional Cancer Center, Ulyanovsk, Russia; 11 Knight Cancer Institute, Oregon Health and Science University, Portland, OR USA

**Keywords:** COVID-19, Genitourinary malignancies, Mortality, Gam-COVID-Vac vaccine

## Abstract

**Background:**

To our knowledge, there is no clinical data pertaining to COVID-19 outcomes and safety of COVID-19 vaccination in Russian patients with genitourinary (GU) malignancies. Aim of our analysis was to describe the characteristics of the COVID-19 infection course as well as preliminary safety and efficacy of Gam-COVID-Vac vaccine in patients with active GU malignancies.

**Methods:**

Patients were retrospectively identified at nine cancer centers in different regions. Patients were included if COVID-19 was diagnosed by a polymerase chain reaction. Data from additional patients with GU cancers who had no positive SARS-CoV-2 RT-PCR test before vaccination and who received two doses of Gam-COVID-Vac (Sputnik V) between 11 February and 31 August 2021 were collected for safety assessment. Anonymized data were collected through an online registry covering demographics, treatments, and outcomes.

**Results:**

The Gam-COVID-Vac vaccine was well tolerated; no grade 3–5 toxicities were reported in 112 vaccinated metastatic GU cancer patients. The most common grade 1 adverse events (81%) were injection site reactions (76%), flu-like illness (68%), and asthenia (49%). Five patients experienced grade 2 chills (4.5%) and 3 patients had grade 2 fever (2.7%). With median follow-up of 6.2 months, two COVID-19 cases were confirmed by RT-PCR test in the vaccine group (of 112 participants; 1.8%). Eighty-eight patients with COVID-19 disease were included in the analysis. The average age as of the study enrollment was 66 (range 39–81) and the majority of patients were male with renal cell carcinoma (RCC). Thirty-six patients (41%) had evidence of metastatic disease, of these 22 patients were receiving systemic therapy. More than half of patients required hospitalization. Fifty-four patients (61%) experienced complications. Sixteen patients who developed COVID-19 pneumonia required mechanical ventilator support. Sixteen patients (18%) died in a median of 23.5 days after the date of COVID-19 diagnosis was established. The 3-month survival rate was 82%. Clinical and/or radiographic progression of cancer during COVID-19 infection or the subsequent 3 months was observed in 10 patients (11.4%).

**Conclusion:**

Patients with GU malignancies are at increased risk of mortality from COVID-19 infection when compared to the general population. Vaccination could be safe in GU cancer patients.

*Trial registration*: retrospectively registered.

## Introduction

Despite global measures to combat COVID-19 pandemic, infection rates are on the rise in Russia [1]. Several previously published analyses have revealed a higher risk of death in cancer patients infected with COVID-19. Available to date clinical evidence supports a higher mortality risk from COVID-19 infection in cancer patients in comparison to patients with no active cancer diagnosis. Several meta-analyses have been published since February 2020 [2–4]. These studies used various search and inclusion criteria, but revealed similar statistically significant association between active cancer diagnosis and COVID-19 mortality (relative risk (RR) 1.66–3.16). Severe complications of COVID-19 infection have also been analyzed in patients with or without active cancer diagnosis. The largest meta-analysis to date based on 18 published individual studies revealed a high relative risk of COVID-19 complications in cancer patients (RR 2.48) [5]. Depending on tumor location, presence of metastases, patient performance status and other factors the severity of COVID-19 infection may vary. Patients with lung cancer and mediastinal metastases had the highest morbidity from COVID-19, up to 25–33% [5, 6]. To our knowledge, there is no clinical data pertaining to COVID-19 outcomes in patients with genitourinary (GU) malignancies in Russia.

GU cancer patients are at risk for the COVID-19 disease. However, the efficacy and safety profiles of vaccines against SARS-CoV-2 in patients with GU cancers are unknown.

To elucidate the characteristics of COVID-19 infection course, rate of death, and preliminary results of safety and efficacy of the COVID-19 vaccine in patients with active GU malignancies we have performed a retrospective analysis.

We conducted retrospective study to determine the COVID-19 infection course, the mortality rate and safety of the COVID-19 vaccine in patients with active GU malignancies.

## Patients and methods

### Vaccinated patients

We collected data from patients with GU cancers who had no positive SARS-CoV-2 RT-PCR test before vaccination and who were vaccinated between 11 February and 31 August 2021. All patients received two doses of Gam-COVID-Vac (Sputnik V) intramuscularly with an interval of 21 days. All participants with available data were included in the safety evaluation according to Common Terminology Criteria for Adverse Events (CTCAE) version 5.0. Currently, follow-up is underway for efficacy evaluation.

### Unvaccinated patients with COVID-19

We invited nine comprehensive oncology clinics situated in different geographical regions of Russia to take part in the data collection and analysis. Our study population included all patients with a diagnosis of GU malignancies (prostate cancer, renal cell carcinoma and bladder cancer) who had a positive SARS-CoV-2 RT-PCR test. Symptomatic and asymptomatic COVID-19 patients were included. Asymptomatic patients were diagnosed by RT-PCR test due to established screening procedures in these regions. Age, gender, histology, or stage of disease were not exclusion criteria. Patients receiving active treatment or on active surveillance were included.

Clinical information was extracted from patients’ records starting on May 2020 and collected until July 2020. Clinical data were divided into four main categories: (1) demographics, (2) history of cancer diagnosis and management, (3) comorbidities, (4) COVID-19 diagnosis, disease course and outcomes. Oncological outcomes were collected through October 24, 2020 for all patients included in this analysis. Factors selected for the original analysis were selected based on available published data.

All data were de-identified and collected centrally for analysis. The study was conducted according to the criteria set by the Declaration of Helsinki and each subject signed an informed consent before participating to the study.

### Statistical analysis

We carried out descriptive statistics characterizing the disease course of COVID-19 in this population of patients with GU malignancies. Demographic and clinical characteristics were presented as rates and median for continuous variables. In multivariate analysis, we included known factors related to mortality risk, such as age (> 60 vs < 60 years of age), gender, ECOG performance status, presence of metastases, treatment modality (surgery vs systemic therapy vs radiation therapy) and cancer type [5]. Patients with missing data were excluded from univariate and multivariate analyses. The proportion was used to calculate the number of adverse events of vaccine and the number of patients infected with SARS-CoV-2.

## Results

### Safety and preliminary efficacy of Gam-COVID-Vac in patients with advanced GU malignancies

The study included 112 vaccinated metastatic GU cancer patients (59 (53%) patients with renal cell carcinoma, 41 (37%) patients with prostate cancer, and 12 (11%) patients with bladder cancer; 77 (69%) patients were male). Median age was 70.6 years (range 29–82). ECOG performance status 0, 1 and 2 were determined in 25 (22%), 71 (64%) and 16 (14%) patients, respectively. Patients were on active systemic therapy (44, 17, 32, 18, and 1 patients received checkpoint inhibitors, tyrosine kinase inhibitors, hormone therapy, chemotherapy, and Radium-223 therapy, respectively). For this interim analysis of safety, data obtained up to September 29, 2021 were analyzed. The most common grade 1 adverse events (81%) were injection site reactions (76%), flu-like illness (68%), and asthenia (49%). Five patients experienced grade 2 chills (4.5%) and three patients had grade 2 fever (2.7%). One patient (0.9%) had transient ischemic attack as a serious non-treatment related adverse event. Vaccine-related hematologic toxicity was not observed in any patient. The reported adverse events of the vaccine did not lead to interruptions, withdrawal or modification of anticancer treatment. In patients receiving immunotherapy, the vaccine did not affect the risk of immune-mediated adverse events.

With median follow-up of 6.2 months, two COVID-19 cases were confirmed by RT-PCR test in the vaccine group (of 112 participants; 1.8%). One patient received cabazitaxel for advanced prostate cancer and another patient received atezolizumab for metastatic urothelial cancer. In both patients, the course of coronavirus infection was mild and was accompanied by a loss of odors and an increase in temperature up to 38C during 8 and 15 days, respectively.

### COVID-19 disease in unvaccinated patients with GU cancers

Eighty-eight patients were included in the analysis. All patients had positive SARS-CoV-2 RT-PCR test. Average age as of the study enrollment was 66 (range 39–81) and the majority of patients were male (Table 1). The most common GU cancer in this study was renal cell carcinoma, followed by prostate cancer and bladder cancer. The majority of patients were diagnosed with localized or locally advanced cancer. At the time of study, enrollment 36 patients (41%) had evidence of metastatic disease, of these 22 patients were receiving systemic therapy and for the remaining 14 patients first- or second-line systemic therapy was planned. The most common therapies included checkpoint inhibitors and targeted therapies. In the majority of patients first-line systemic therapy was administered to patients on average 7 days (range 0–11) prior to the date of positive SARS-CoV-2 RT-PCR test (Table 1). Three patients elected to continue with prescribed therapy without signs of increased toxicity. Forty-six patients (52%) underwent surgery no later than 14 days prior to the date of positive SARS-CoV-2 RT-PCR test. Fifteen patients (17%) were receiving radiation therapy at the time of positive SARS-CoV-2 RT-PCR test.

**Table 1:** Baseline and treatment characteristics of 88 patients

Number of patients	88
Age (years), median (range)	63.5 (39–81)
Gender	
Male	64 (73)
Female	24 (27)
Histology, *N* (%)	
Renal cell carcinoma	38 (43)
Prostate cancer	26 (30)
Urothelial cancer	24 (27)
Cancer Stage (UICC, 8th ed.), *N* (%)	
I	16 (18)
II	16 (18)
III	20 (23)
IV	36 (41)
Distant metastases, *N* (%)	
No	52 (59)
Yes:	36 (41)
Lung	27 (31)
Liver	14 (16)
Lymph nodes	13 (15)
Bone	10 (11)
Pleura	5 (6)
Brain	1 (1)
Pancreas	1 (1)
Surgical treatment at the time of COVID-19	
Before surgery, *N* (%)	1 (1)
After surgery, *N* (%)	46 (52)
Time from performed surgery to COVID-19, median (days, range)	6.5 (2–14)
Radiation therapy at the time of COVID-19, *N* (%)	15 (17)
Systemic therapy at the time of COVID-19 infection, *N* (%)	
Patients during first-line therapy	18 (21)
Patients during subsequent therapy	4 (5)
Checkpoint inhibitors	8 (9)
Targeted therapy	7 (8)
Combination of checkpoint inhibitors and	1 (1)
Targeted agent	
Chemotherapy/gonadotropin releasing hormone analogs	6 (27)
Not started (in case of initial planning)	14 (16)
Interrupted (if the patient was on therapy)	19 (9)
Continued	3 (3)

The most common symptoms of COVID-19 infection were fever, fatigue, cough and dyspnea (Table 2). Fifteen patients (17%) were asymptomatic. More than half of patients required hospitalization. Median time from onset of symptoms or date of diagnosis and hospitalization was six days (range 2–10). Fifty-four patients (61%) experienced complications. Pneumonia was the most common complication, and dyspnea was the most common symptom of disease. Sixteen patients who developed COVID-19 pneumonia required mechanical ventilator support (Table 2). Oxygen support was provided to 12 patients (14%). The remaining patients had mild symptoms and remained isolated at home. ECOG performance status deteriorated during COVID-19 infection in half of study patients.

**Table 2:** COVID-19 clinical presentation and outcomes

Common symptoms of COVID-19, *N* (%)	
Fever	60 (68)
Fatigue	48 (53)
Cough	39 (44)
Dyspnea	25 (28)
Complications of COVID-19, *N* (%)	
Pneumonia	44 (61)
Respiratory failure	10 (11)
Multiple organ failure	8 (9)
Acute respiratory distress syndrome	6 (7)
Pancreatitis	2 (2)
Abnormal coagulation	2 (2)
Anemia	1 (1)
Frequency of ECOG performance status deterioration during COVID-19, %	48 (55)
Course of COVID-19	
Mild	46 (52)
Noncritical care hospitalization	14 (16)
Critical care hospitalization (oxygen support)	12 (14)
Critical care hospitalization (mechanical ventilation)	16 (18)
Duration of COVID-19, median (days)	19.8
Length of hospitalization, median (days)	22.0
Antiviral/COVID-19 therapy, *N* (%)	42 (48)
Hydroxychloroquine	31 (35)
Azithromycin	17 (19)
Dexamethasone	17 (19)
Favipiravir	2 (2)
Ritonavir + Lopinavir	1 (1)
COVID-19 disease outcome	
Convalescence, *N* (%)	72 (82)
Death, *N* (%)	16 (18)
3-month overall survival, %	82

The median duration of COVID-19 infection was 19.8 days; the median duration of hospitalization was 22.0 days. Sixteen patients (18%) died in a median of 23.5 days after the date of COVID-19 diagnosis was established. Three-month survival rate was 82%. In the univariate analysis, advanced age (OR 2.17, 95% CI 1.18–4.02), renal cell carcinoma (OR 3.72, 95% CI 2.1–8.65), presence of distant metastases (OR 6.15, 95% CI 3.39–10.5), receipt of systemic therapy (OR 2.73, 95% CI 1.11–4.29), ECOG performance status 2 or higher on the date of positive SARS-CoV-2 RT-PCR test (OR 6.1, 95% CI 3.61–12.07) were factors associated with higher risk of mortality. In the multivariate analysis (Table 3), presence of distant metastases (OR 3.29, 95% CI 1.77–6.21) and ECOG performance status 2 or higher on the date of positive SARS-CoV-2 RT-PCR test (OR 5.36, 95% CI 2.84–11.76) remained predictors of higher mortality. The median time of treatment interruption, defined as the interval between the last day of active treatment to the date of return to the same treatment modality, due to COVID-19 diagnosis was 38.9 days (range 18–64).

**Table 3:** Multivariable model of factors associated with death

Factor	Odds ratio (95% CI)
Age > 60 years (vs ≤ 60 years)	0.79 (0.41–1.92)
Renal cell carcinoma (vs other types)	1.18 (0.55–2.11)
Distant metastases (vs localized/locally advanced disease)	3.29 (1.77–6.21)
ECOG performance status ≥ 2 (vs 0–1)	5.36 (2.84–11.76)
Systemic therapy (vs surgery vs radiation therapy)	1.15 (0.27–8.90)

With median follow-up of 3.5 months, clinical and/or radiographic progression of cancer during COVID-19 infection was observed in 10 patients (11.4%). Anticancer treatment was interrupted in all of these patients. Characteristics of these patients are summarized in Table 4. These patients had the diagnosis of renal cell carcinoma (*n* = 4), prostate cancer (*n* = 4) and bladder cancer (*n* = 2). The cancer stage (UICC, 8th edition) distribution among these 10 patients was as follows: Stage I (*n* = 1), Stage III (*n* = 2) and Stage IV (*n* = 7). Among these 10 patients with cancer progression, 6 patients required mechanical ventilator support or oxygen support, 4 patients were admitted to non-intensive care unit in the hospital. All these 10 patients convalesced over a median of 28.8 days.

**Table 4:** Clinical and/or radiographic progression of cancer during COVID-19 disease

Number of patients	10
Age (years), mean(range)	77.4 (39–81)
Gender	
Male	4(40)
Female	6 (60)
Cancer Stage (UICC, 8th ed.), *N* (%)	
I	1 (10)
II	0 (0)
III	2 (20)
IV	7 (70)
Histology, *N* (%)	
Renal cell carcinoma	4 (40)
Prostate cancer	4 (40)
Urothelial cancer	2 (20)
Course of COVID-19, *N* (%):	
Mild	0 (0)
Mechanical ventilation	2 (20)
Noncritical care hospitalization	4 (40)
Critical care hospitalization (oxygen support)	4 (40)
Duration of COVID-19, median (days)	28.8
Death as a COVID-19 disease outcome, *N* (%)	4 (40)

## Discussion

This is the largest to date retrospective analysis of clinical outcomes among GU cancer patients diagnosed with COVID-19 infection in Russia. Based on our analysis of 88 patients with active GU malignancies, we estimate the mortality risk of 18% in this group of patients, which is similar to the entire population of cancer patients, as previously reported at the annual 2020 ASCO Annual Meeting [7]. In the multivariate analysis, we have identified presence of distant metastases and poor baseline performance status as factors associated with higher COVID-19 mortality. We were surprised that age did not remain a predictive factor in our analysis. Subsequent analysis with expansion of patient population will be necessary to determine whether age remains a poor predictor of survival from COVID-19 infection in this group of patients with GU malignancies, as well as clarify the role of active systemic therapy in these patients. Heterogeneity of patients and relatively small sample size are the main limitations of our study.

One-half of patients enrolled on our study experienced mild course of COVID-19 disease, which translated into a relatively high 3-month survival rate of 82%. The duration of COVID-19 disease —as well as duration of hospitalization—are in line with previously reported numbers [4–7]. At the same time, one-half of patients in our analysis experienced severe symptoms, with pneumonia as the most common clinical presentation. A third of patients with severe COVID-19 infection required oxygen support or mechanical ventilation. The other 16% were hospitalized for other medical reasons.

Oncological care was interrupted for a period of 18 to 64 days in our study. We cannot exclude that this interruption at least in part contributed to disease progression in 11% of these patients over the course of subsequent 3 months. At the present time, the safety of observation replacing active management of metastatic GU malignancies during the COVID-19 pandemic remains open and requires additional assessment as well as region-specific policy development. This is particularly relevant for patients with metastatic disease. Nevertheless, 3 patients with GU malignancies infected with COVID-19 elected to continue with their on-going oncological therapies without experiencing adverse outcomes in our study. Thus, with very limited information and no comparison on patients' health conditions between groups with or without anticancer treatment, no solid conclusion should be generated. This limited experience goes against the guidelines and recommendations issued by national and international associations, which advocate for cessation of active anticancer therapies until resolution of COVID-19 infection [8–13]. However, COVID-19 vaccination of cancer patients may change the approach to continuing active systemic treatment. Vaccines can be safe and effective [14]. More data are needed on the use of Russian vaccines in GU cancer patients.

## Conclusion

In conclusion, patients with GU malignancies experiencing COVID-19 have higher mortality associated with the presence of metastatic disease and ECOG performance status. The patients who did not interrupt their treatment, did not have excess in complications of COVID-19. Patients who did have an interruption of their treatment, however, might have worse outcome in the long-term follow-up, and we need to be careful to interrupt treatments. Vaccination should be used in cancer patients under the active supervision of an oncologist.

## Data Availability

Not applicable.

## Abbreviations

CI: Confidence interval
CTCAE: Common Terminology Criteria for Adverse Events
ECOG: Eastern Cooperative Oncology Group
GU: Genitourinary
OR: Odds ratio
RR: Relative risk
RT-PCR: Reverse transcription polymerase chain reaction
